# “Iliacus muscle abscess as an unexpected cause of posterior hip pain in a healthy young adult female”: a case report

**DOI:** 10.1186/s12245-024-00668-4

**Published:** 2024-07-17

**Authors:** Caleb Weihao Huang, Mathew Yi Wen Yeo

**Affiliations:** Acute & Emergency Care, Khoo Teck Puat Hospital, 90 Yishun Central, Singapore, Singapore

**Keywords:** Iliacus abscess, Iliopsoas abscess, Hip pain

## Abstract

**Background:**

Iliacus muscle abscess is an uncommon but potentially life-threatening condition that can present with nonspecific symptoms, posing diagnostic challenges. This case report highlights the importance of considering iliopsoas abscess in patients presenting with fever and hip pain, especially in the absence of obvious risk factors or penetrating trauma. The novelty of this case lies in its atypical presentation mimicking a respiratory viral infection and musculoskeletal injury, impeding accurate diagnosis and appropriate management.

**Case Presentation:**

A previously healthy 21-year-old female who had a mechanical fall 3 weeks prior presented with fever, right hip pain, and respiratory symptoms, initially suggestive of a respiratory infection and musculoskeletal injury. However, initial investigations revealing a markedly high C-reactive protein (CRP) concentration prompted further computed tomography (CT) imaging of her abdomen and pelvis, which uncovered an iliopsoas abscess presumably stemming from antecedent trauma. Subsequent CT guided aspiration along with culture-sensitive antibiotics led to successful treatment and resolution of her symptoms.

**Conclusions:**

This case emphasizes the importance of considering iliopsoas abscess as a possible differential, even in young patients without typical risk factors. Markedly elevated inflammatory markers such as CRP concentrations can serve as a vital indicator, directing attention towards the possibility of septicemia or the presence of an occult abscess, facilitating prompt imaging and accurate diagnosis.

## Introduction

Iliacus muscle abscess is uncommon and may arise from hematogenous seeding from a distant site (primary abscess) or from a direct spread from an adjacent structure (secondary abscess). Risk factors for a primary muscle abscess include intravenous drug use, tuberculosis, local recent surgery, trauma, or underlying immunosuppression such as diabetes mellitus, human immunodeficiency virus or malignancy [1]. A secondary muscle abscess stems from direct spread from an adjacent infected structure such as from appendicitis, diverticulitis, or neighboring spondylodiscitis [2].

Located next to the psoas major, the iliacus muscle merges with the psoas and is given the common name iliopsoas. Timely diagnosis of an iliopsoas abscess is vital as its mortality rate ranges between 5 and 15% [3]. However, its presentation of nonspecific clinical symptoms such as fever, hip and lumbar pain [4], poses a significant diagnostic challenge. In this case report, we detail the clinical presentation of a young female patient exhibiting fever, right-sided hip pain, and symptoms suggestive of respiratory infection, which initially served as a red herring in the diagnostic process.

## Case presentation

A previously well 21-year-old female patient presented to the emergency department for 6 days of fever with a highest temperature of 39 °C, associated with right hip pain, sore throat, rhinorrhea, and nausea. She visited the outpatient clinic 5 days ago for right hip pain after standing up from a seated position during a parade rehearsal. Three weeks earlier, she experienced a fall backward, landing on her buttocks due to abrupt braking of the bus she was aboard. Since then, her right hip pain has persisted, being worse in the supine position, and affecting her sleep despite analgesia. She reports no vaginal discharge or bleeding.

On arrival at the emergency department, she had a temperature of 38.4 °C, heart rate of 113 beats per minute, blood pressure of 118/73 mmHg, respiratory rate of 18 breaths per minute, and an oxygen saturation of 100% on room air. She exhibited visible lethargy and shivering, along with dry mucous membranes. Tenderness was noted over the upper and outer quadrant of her right gluteus with no visible swelling, bruising or overlying skin changes. There was no midline or paravertebral tenderness over her lumbar region. Due to the pain over the right hip, she had trouble sitting upright. Examination of her throat revealed an erythematous pharyngeal wall with an ulcer noted over the uvula and left lateral wall of the mouth. Uvula was central and tonsils were not enlarged.

### Investigations and treatment

A broad infectious workup was pursued. Initially investigations showed the white blood cell (WBC) count to be 3,560 / µL which increased to 18,240 / µL (neutrophils 78%) the next day. Initial C-reactive protein (CRP) concentration was 323.6 mg/L (reference < 5 mg/L) and procalcitonin concentration of 2.5 ng/mL (reference < 2 ng/mL). Chest and soft tissue neck X-rays did not show any pneumonia, epiglottitis, or prevertebral soft tissue thickening. Urine dipstick was negative for leukocytes, blood, and nitrites. Liver function test showed mildly raised transaminases. Aspartate transaminase (AST) was 39 U/L (reference < 30 U/L), alanine transaminase (ALT) was 41 U/L (reference < 36 U/L), ALP was 178 U/L (reference < 104 U/L). Blood cultures were also obtained, and the patient was started on empirical intravenous (IV) Augmentin.

**Fig. 1:**
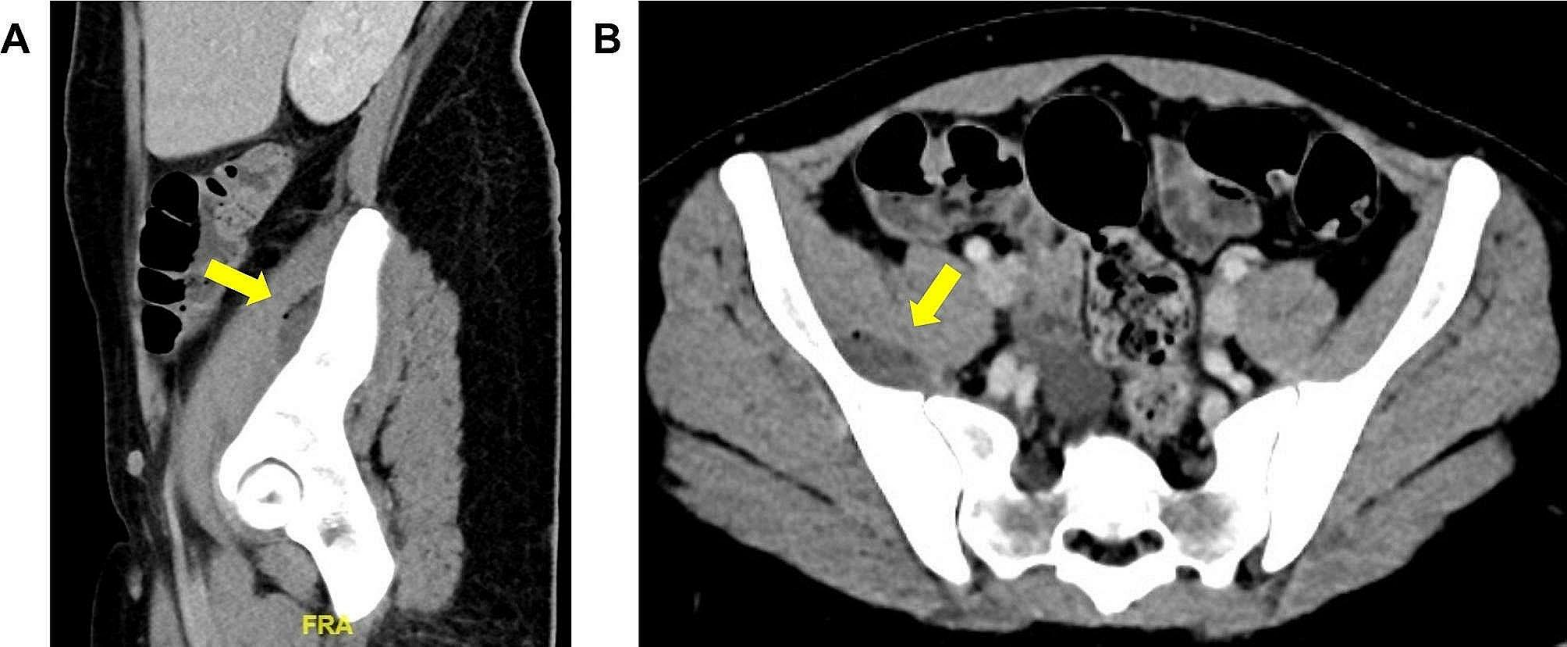
Computed tomography imaging with contrast, (**A**) sagittal and (**B**) axial views. Arrow: a gas-containing, faintly rim-enhancing fluid collection (17 ± 8 Hounsfield unit) at the deep aspect of the right iliacus muscle overlying the right sacroiliac (SI) joint measuring 3.4 × 1.2 × 3.9 cm

Due to the markedly raised inflammatory markers and right hip pain that seemed out of proportion, a computed tomography (CT) of the abdomen and pelvis was performed, revealing a gas-containing, faintly rim-enhancing fluid collection at the deep aspect of the right iliacus muscle overlying the right sacroiliac (SI) joint, measuring 3.4 × 1.2 × 3.9 cm (Fig. 1). A thin-walled 4.5 × 3.8 cm right adnexal cystic lesion, periportal edema in the liver, as well as patchy consolidations and ground-glass opacities were also noted in the partially imaged lower lobes of both lungs.

**Fig. 2:**
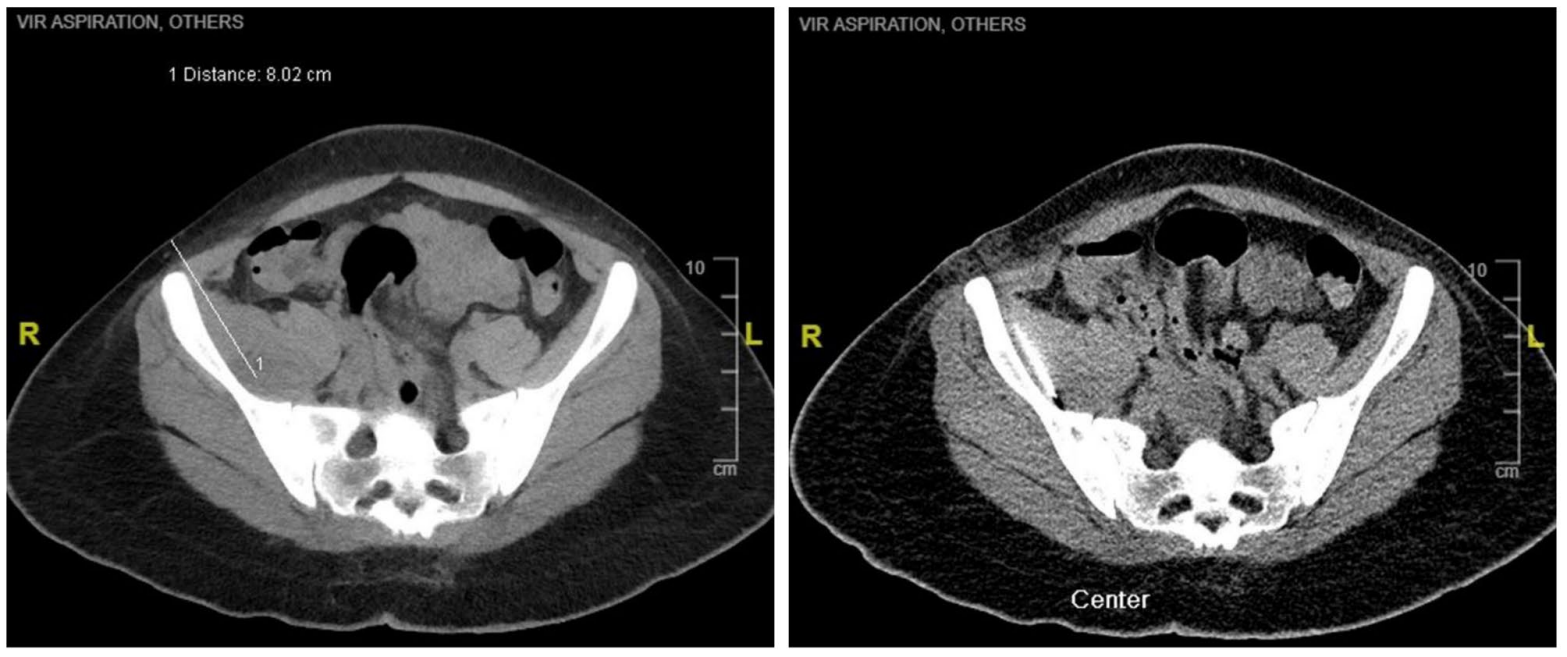
Axial view of computed tomography guided aspiration of the right iliacus muscle abscess located approximately 8.02 cm from the skin

The patient was referred to Orthopedics and underwent CT guided aspiration of the right iliacus collection (Fig. 2). Thirty ml of purulent fluid was aspirated which grew Klebsiella aerogenes, in line with the results of her blood culture. Acid fast bacilli (AFB) and fungal smear from the collection were negative. She was also screened for Chlamydia trachomatis, Neisseria gonorrhoea, Syphillis, Hepatitis A, B and C which were all negative. She received initial treatment with IV Augmentin, later transitioning to IV Meropenem and Ceftriaxone, and subsequently to Ciprofloxacin and Metronidazole following consultation with infectious disease specialists. Post-aspiration of the right iliacus collection, her inflammatory markers trended downwards and the pain in her hip significantly improved. She remained on oral Ciprofloxacin for a duration of 6 weeks, scheduled for a follow-up CT scan of the abdomen and pelvis 6 weeks post-discharge, along with a subsequent review by infectious disease specialists. She was also given an outpatient follow-up with Gynecology as an ultrasound of the pelvis revealed cystic lesions in the right ovary, and a non-specific vague region of heterogenous echogenicity in the anterior fundus of the uterus suspicious of a fibroid.

## Discussion

Missing an iliacus or iliopsoas abscess can be potentially fatal due to its high morbidity and mortality rates [5]. However, it is an easily overlooked infectious disease due to its nonspecific presentation and insidious onset. This is especially so in a patient who has experienced antecedent trauma, or is dealing with an intercurrent illness, both of which could manifest as hip pain or a backache.

Our patient, who had no significant risk factors, experienced antecedent trauma after falling on her buttocks 3 weeks prior. Muscle strain or hematoma formation following trauma or strenuous exercise [6] are more commonly observed in such scenarios, though the rarity of deep soft tissue abscess formation secondary to non-penetrating trauma has been described in an early report [7].

Inflammatory markers like WBC count and CRP concentration are routinely employed to ascertain the presence of an active infection or inflammatory condition. Despite exhibiting classic symptoms of herpangina including high fever, respiratory symptoms and oral ulcers, our patient’s elevated CRP level of 323.6 mg/L instigated further investigation into alternative bacterial infection sources. A prior study has established the effectiveness of CRP concentration as a sensitive and specific predictor of bacterial infection in individuals with concurrent influenza-like illness [8], highlighting its pivotal role in the diagnostic scenario presented in this report.

Diagnosis of an iliacus abscess can be confirmed by means of advanced imaging such as a CT, as seen in this patient. However, a limitation of CT scans is the potential for false negatives in cases where the abscess exhibits low attenuation or does not contain air [2]. In such instances, magnetic resonance imaging (MRI) may be a valuable alternative as it has demonstrated greater sensitivity than CT in detecting intra-abdominal abscesses, and is more accurate in delineating inflammatory changes, extending beyond the abscess site [9].

The causative agent in our patient was Klebsiella aerogenes, identified through blood culture, causing septicemia, and confirmed by abscess drainage for which the patient underwent. Source control by aspiration drainage is commonly used for a single abscess, while open surgery is recommended for complex abscesses [10, 11]. Culture sensitive guided antibiotics were subsequently used leading to the patient’s prompt recovery and discharge.

In conclusion, this case underscores the importance of thorough investigation in individuals presenting with fever accompanied by back or hip pain. Even among young adults devoid of underlying illnesses or seemingly benign antecedent traumas, an iliacus abscess may still manifest. The use of CRP as an inflammatory marker also aids in determining the necessity for timely advanced imaging, such as CT or MRI studies, facilitating accurate diagnosis and prompt treatment.

## Data Availability

No datasets were generated or analysed during the current study.
